# The duration of the interpregnancy interval in multiparous women and maternal weight gain between pregnancies: findings from a UK population-based cohort

**DOI:** 10.1038/s41598-019-45595-0

**Published:** 2019-06-24

**Authors:** Nida Ziauddeen, Paul J. Roderick, Nicholas S. Macklon, Nisreen A. Alwan

**Affiliations:** 1 School of Primary Care and Population Sciences, Faculty of Medicine, University of Southampton, Southampton, SO16 6YD UK; 2 Department of Obstetrics and Gynaecology, Zealand University Hospital, Roskilde, Denmark; 3 London Women’s Clinic, 113-115 Harley Street, London, W1G 6AP UK; 4 NIHR Southampton Biomedical Research Centre, University of Southampton and University Hospital Southampton NHS Foundation Trust, Southampton, SO16 6YD UK

**Keywords:** Epidemiology, Risk factors

## Abstract

Maternal obesity in pregnancy increases the risk of adverse long-term health outcomes in both mother and offspring. A population-based cohort of prospectively collected routine antenatal healthcare data collected between January 2003 and September 2017 at University Hospital Southampton, UK was utilised to investigate the association between duration of interpregnancy interval between successive pregnancies and gain in maternal body mass index by the start of the next pregnancy. Records of 19362 women with two or more consecutive singleton live births were analysed. Two-thirds had gained weight when presenting to antenatal care for their subsequent pregnancy with 20% becoming overweight/obese. Compared to an interval of 24–35 months, an interval of 12–23 months was associated with lowest risk of weight gain (adjusted RR 0.91, 99% CI 0.87 to 0.95, p < 0.001) and ≥36 months with greatest risk (adjusted RR 1.11, 99% CI 1.07 to 1.15, p < 0.001) for the first to second pregnancy. This study shows that most multiparous women start their pregnancy with a higher weight than their previous one. An interval of 12–23 months is associated with the lowest risk of starting the second pregnancy with a higher body weight accounting for age. In countries with high prevalence of maternal obesity, birth spacing may merit exploration as a factor impacting on perinatal morbidity.

## Introduction

Pregnancy is a period of metabolic and behavioural changes, the effects of which last beyond the immediate pregnancy for both mother and child^1^ thus affecting subsequent children. Biological and behavioural changes on childbearing can lead to weight gain and can alter a woman’s weight trajectory^2^. Maternal obesity is a key predictor of maternal and fetal pregnancy outcomes as well as long-term health outcomes in the mother and child such as diabetes and cardiovascular disease^3^. Overweight and obesity prevalence has been increasing over the last few decades with data from the Health Survey for England 2015 indicating that an average of 52.1% of women aged 16 to 54 years are overweight or obese^4^. This rise in obesity in women of childbearing age and its associated effects on maternal and offspring health^3^ make maternal weight change between pregnancies an important consideration as this could modify risk of subsequent offspring.

Women who have given birth are at higher risk of developing obesity than women who have not^5^. Additionally, women with excess gestational weight gain who failed to lose pregnancy weight by six months postpartum were at increased risk of subsequent obesity^6^. Although overweight and obesity in nulliparous women is associated with increased risk of adverse outcomes^7^, evidence on association with increased risk of postpartum weight retention is conflicting^8–10^ with a review concluding that gestational weight gain rather than pre-pregnancy body mass index (BMI) determines postpartum weight retention^11^. A systematic review reported that postpartum weight follows a steep decrease in the first three months followed by a continuous decrease until 12 months following which an increase in weight was reported. However, this was only assessed in two cohorts^2^. Post-partum weight retention is variable with women on average retaining 0.5 to 3 kg, however a substantial number (12–20%) retain a considerable amount of weight^12^. Approximately two-thirds of women presenting for antenatal care for a second pregnancy in Ireland an average of 18 months after delivery had gained weight with 20% in a higher compared to 5.8% in a lower BMI category than the first pregnancy^13^.

The World Health Organization technical consultation on birth spacing in 2005 recommended an interval of 2 years or more however evidence on maternal obesity as an outcome was not considered^14^. One of the major concerns with a short interval is maternal nutritional depletion because of inadequate time to recover from one pregnancy before entering the next^15^. In the US, nearly a third of second order or higher births were conceived within 18 months of the previous with 5% conceived within six months^16^. There is evidence that interpregnancy interval gets shorter as maternal age at first pregnancy increases, with women who delay the start of childbearing to ≥35 years having increased odds of intervals less than six months^17^. Data from 1969–2006 in Switzerland showed that maternal age at first pregnancy had increased from 25.0 to 30.1 years with shorter intervals between pregnancies^18^. Short (<18 months) and long (>59 months) intervals between pregnancies has been associated with increased risk of adverse perinatal outcomes^19^ such as preterm birth, low birth weight and small-for-gestational age^19,20^.

Weight retention is highest after the first pregnancy^21^, and gestational weight gain and postpartum weight retention in subsequent pregnancies follow a similar pattern to the first^8^. Analysis of a retrospective cohort of 37178 women with three pregnancies in Canada found that women with short interpregnancy intervals (<12 months compared to 18–23 months) were more likely to enter the subsequent pregnancy obese^22^. However, BMI at the start of the previous pregnancy and socioeconomic status were not taken into account.

To our knowledge, no previous epidemiological studies have examined gain in maternal BMI in multiparous women in relation to birth spacing. The aim of this study was therefore to examine, in a population-based cohort of antenatal healthcare data in the South of England, patterns of gain in first-trimester maternal BMI, and examine its association with the length of the interpregnancy interval between consecutive live births.

## Results

**Figure 1:**
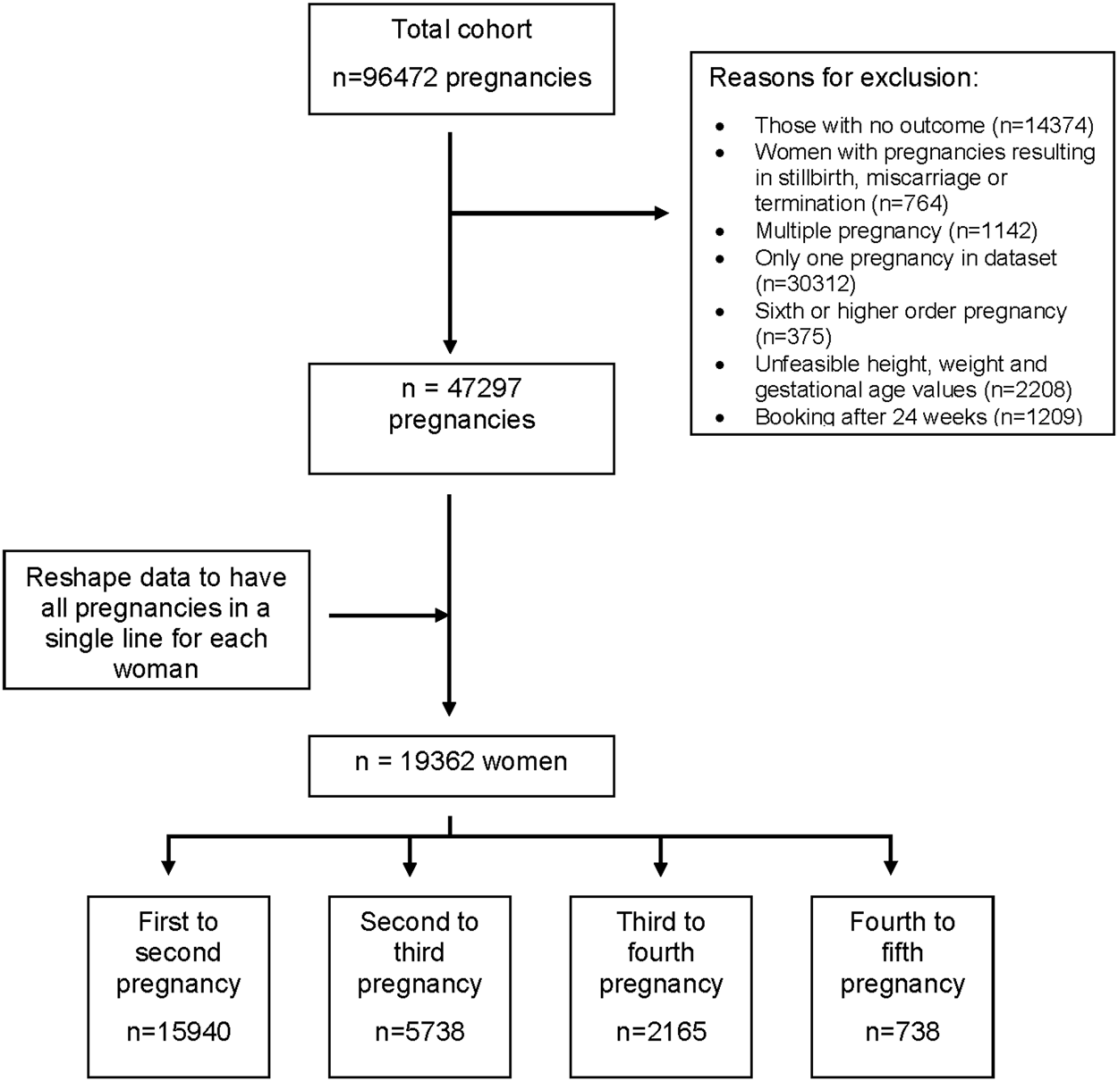
Flow diagram showing the data preparation process.

The main sample consisted of 19362 women with at least two consecutive live birth pregnancies (Fig. 1). Of the 15940 women who had their first two pregnancies in the dataset, 12636 women only had first two, 2654 had three, 530 had four and 120 had five consecutive pregnancies. A further 1884 women had their second to third, 430 second to fourth, 136 second to fifth, 758 third to fourth, 207 third to fifth and 7 fourth to fifth pregnancies. A description of the sample characteristics by pregnancy order is shown in Table 1. Mean maternal BMI at first pregnancy was 24.6 kg/m^2^ (standard deviation 5.0) and increased with pregnancy order. Overweight and obesity in the sample increased with higher order pregnancies with 13.0% obese at first pregnancy to 31.6% obese at fifth pregnancy. The proportion of women who stopped smoking when pregnancy was confirmed was highest in the first pregnancy and decreased in subsequent pregnancies. The proportion of women who continued smoking through pregnancy was highest in later pregnancies. Women with college education or lower tended to have higher number of pregnancies and higher BMI. There was a slight shift in ethnic distribution from first to higher order pregnancies with a decrease in the proportion of White women and an increase in the proportion of Asian and Black/African/Caribbean women.

**Table 1:** Pregnancy characteristics by gestational order for period of January 2003 - September 2017, University Hospital Southampton NHS Foundation Trust, Southampton, Hampshire, England.

	First pregnancy	Second pregnancy	Third pregnancy	Fourth pregnancy	Fifth pregnancy
N	15940	18954	6844	2533	738
Maternal age (mean ± SD)	25.9 ± 5.5	28.6 ± 5.4	29.3 ± 5.0	30.3 ± 4.9	31.6 ± 4.8
Timing of first booking appointment, weeks (mean ± SD)	11.3 ± 2.7	11.2 ± 2.5	11.5 ± 2.8	11.8 ± 3.1	12.0 ± 3.3
Maternal BMI (mean ± SD)	24.6 ± 5.0	25.8 ± 5.6	26.5 ± 6.0	27.3 ± 6.2	28.2 ± 6.6
**Maternal BMI (%**, **99% CI)**					
Underweight (<18.5)	3.9 (3.5 to 4.3)	2.9 (2.6 to 3.2)	2.5 (2.0 to 3.0)	2.1 (1.5 to 3.0)	1.5 (0.6 to 3.1)
Normal weight (18.5 to 24.9)	59.2 (58.2 to 60.2)	51.6 (50.6 to 52.5)	46.1 (44.5 to 47.7)	41.1 (38.6 to 43.7)	36.2 (31.7 to 40.9)
Overweight (25.0 to 29.9)	23.9 (23.0 to 24.7)	26.6 (25.8 to 27.5)	28.7 (27.3 to 30.2)	28.4 (26.1 to 30.8)	30.8 (26.5 to 35.3)
Obese (≥30.0)	13.0 (12.4 to 13.7)	18.9 (18.2 to 19.7)	22.7 (21.4 to 24.0)	28.3 (26.0 to 30.7)	31.6 (27.2 to 36.2)
**Maternal smoking status (%**, **99% CI)**					
Never smoked/quit	53.3 (52.3 to 54.4)	57.5 (56.5 to 58.4)	50.8 (49.3 to 52.4)	47.6 (45.0 to 50.2)	45.3 (40.5 to 50.1)
Stopped >1 year before conceiving	12.0 (11.4 to 12.7)	16.2 (15.5 to 16.9)	14.7 (13.6 to 15.8)	12.7 (11.1 to 14.5)	11.1 (8.3 to 14.4)
Stopped <1 year prior to conceiving	7.3 (6.8 to 7.8)	4.1 (3.8 to 4.5)	4.2 (3.6 to 4.8)	3.2 (2.4 to 4.2)	5.4 (3.5 to 7.9)
Stopped when pregnancy confirmed	12.1 (11.4 to 12.7)	7.4 (6.9 to 7.9)	7.5 (6.7 to 8.3)	7.6 (6.3 to 9.1)	6.4 (4.3 to 9.0)
Continued smoking	15.3 (14.6 to 16.0)	14.8 (14.2 to 15.5)	22.8 (21.5 to 24.2)	28.9 (26.6 to 31.3)	31.8 (27.5 to 36.4)
**Maternal education (%**, **99% CI)**					
Secondary (GCSE) or under	23.7 (22.9 to 24.6)	24.9 (24.1 to 25.7)	36.3 (34.8 to 37.8)	45.9 (43.3 to 48.5)	51.8 (47.0 to 56.5)
College (A levels)	43.0 (42.0 to 44.0)	43.2 (42.3 to 44.1)	44.0 (42.5 to 45.6)	41.8 (39.3 to 44.4)	41.7 (37.1 to 46.5)
University degree or above	33.3 (32.3 to 34.3)	31.9 (31.0 to 32.8)	19.7 (18.5 to 21.0)	12.3 (10.7 to 14.1)	6.5 (4.4 to 9.2)
**Maternal employment (%**, **99% CI)**					
Employed	80.0 (79.1 to 80.8)	64.0 (63.1 to 64.9)	45.4 (43.8 to 46.9)	28.8 (26.5 to 31.2)	20.5 (16.8 to 24.5)
Unemployed	15.7 (14.9 to 16.4)	34.3 (33.4 to 35.1)	52.3 (50.7 to 53.9)	68.7 (66.3 to 71.1)	77.5 (73.3 to 81.3)
In education	4.0 (3.6 to 4.4)	1.1 (0.9 to 1.3)	1.3 (1.0 to 1.7)	1.3 (0.8 to 2.0)	1.1 (0.3 to 2.5)
Not specified	0.4 (0.3 to 0.6)	0.7 (0.5 to 0.9)	1.0 (0.7 to 1.4)	1.2 (0.7 to 1.9)	0.9 (0.3 to 2.3)
**Ethnicity (%**, **99% CI)**					
White	86.9 (86.1 to 87.5)	85.7 (85.0 to 86.3)	82.6 (81.4 to 83.7)	81.2 (79.1 to 83.1)	81.7 (77.8 to 85.2)
Mixed	1.2 (1.0 to 1.4)	1.2 (1.0 to 1.5)	1.2 (1.0 to 1.6)	1.4 (0.9 to 2.1)	1.9 (0.8 to 3.6)
Asian	5.8 (5.3 to 6.3)	6.3 (5.8 to 6.8)	9.4 (8.5 to 10.4)	10.0 (8.5 to 11.6)	9.6 (7.0 to 12.8)
Black/African/Caribbean	1.4 (1.2 to 1.7)	1.7 (1.5 to 1.9)	2.5 (2.1 to 3.1)	3.4 (2.5 to 4.4)	3.4 (1.9 to 5.5)
Chinese	0.6 (0.4 to 0.7)	0.5 (0.4 to 0.7)	0.3 (0.1 to 0.5)	0.3 (0.1 to 0.7)	0.1 (0.0 to 1.0)
Other	1.0 (0.8 to 1.2)	1.2 (1.0 to 1.4)	1.4 (1.1 to 1.8)	1.5 (0.9 to 2.2)	1.8 (0.8 to 3.4)
Not specified	3.2 (2.9 to 3.6)	3.4 (3.1 to 3.8)	2.6 (2.1 to 3.1)	2.3 (1.6 to 3.2)	1.5 (0.6 to 3.1)

Table 2 summarizes the interpregnancy interval and change in maternal BMI between consecutive pregnancies. Median interpregnancy interval followed a u-shaped pattern and was shortest from first to second pregnancy, increased from second to third pregnancy but decreased for subsequent pregnancies and was similar to the interval between first to second pregnancy. However, the proportion of women with an interval of 0–11 months between pregnancies increased from 17.5% in the first to second pregnancy to 28.5% in the fourth to fifth pregnancy. Between 47–52% of women had intervals of 2 years or more between pregnancies. The median overall change in maternal BMI from first to second pregnancy was 0.9 kg/m^2^ (interquartile range IQR −0.4 to 2.4) however the change in women who lost weight was 1.0 kg/m^2^ (IQR −1.9 to −0.5) and in women who gained weight, it was 1.8 kg/m^2^ (IQR 0.9 to 3.4). The change remained similar across pregnancies with approximately two-thirds of women having gained weight when presenting for antenatal care for the subsequent pregnancy. Over a fifth were in a higher BMI category by start of the next pregnancy with 1–2% having moved two BMI categories (for example, normal weight to obese).

**Table 2:** Change in maternal body mass index (BMI) measured at the first antenatal visit between consecutive pregnancies by gestational order.

	First to second pregnancy	Second to third pregnancy	Third to fourth pregnancy	Fourth to fifth pregnancy
N	15940	5738	2165	738
Interpregnancy interval, months (median, IQR)	22.9 (14.6 to 35.5)	25.0 (14.0 to 43.1)	22.6 (12.3 to 40.7)	22.9 (10.8 to 41.1)
**Interpregnancy interval**, **categorised (%**, **99% CI)**				
0–11 months	17.5 (16.8 to 18.3)	19.7 (18.4 to 21.1)	24.7 (22.3 to 27.1)	28.5 (24.3 to 32.9)
12–23 months	35.3 (34.3 to 36.3)	28.2 (26.7 to 29.8)	28.5 (26.0 to 31.0)	23.8 (19.9 to 28.1)
24–35 months	23.1 (22.2 to 23.9)	18.7 (17.4 to 20.0)	16.7 (14.7 to 18.9)	18.0 (14.5 to 21.9)
36 months or more	24.1 (23.3 to 25.0)	33.4 (31.8 to 35.0)	30.2 (27.6 to 32.8)	29.7 (25.4 to 34.2)
24 months or more	47.2 (46.2 to 48.2)	52.1 (50.4 to 53.8)	46.9 (44.1 to 49.7)	47.7 (42.9 to 52.5)
**Direction of change of maternal BMI (%**, **99% CI)**				
No change	2.9 (2.6 to 3.3)	2.9 (2.3 to 3.5)	3.2 (2.3 to 4.3)	3.5 (2.0 to 5.7)
Lost BMI units	31.3 (30.3 to 32.2)	31.8 (30.2 to 33.4)	31.7 (29.1 to 34.3)	27.4 (23.2 to 31.8)
Gained BMI units	65.8 (64.9 to 66.8)	65.3 (63.7 to 66.9)	65.1 (62.4 to 67.8)	69.1 (64.5 to 73.4)
Change in maternal BMI (median, IQR)	0.9 (−0.4 to 2.4)	0.9 (−0.4 to 2.5)	0.9 (−0.4 to 2.8)	1.3 (−0.2 to 2.8)
Change in maternal BMI in women who lost weight	−1.0 (−1.9 to −0.5)	−1.2 (−2.2 to −0.5)	−1.3 (−2.4 to −0.6)	−1.1 (−2.3 to −0.6)
Change in maternal BMI in women who gained weight	1.8 (0.9 to 3.4)	1.9 (0.9 to 3.4)	2.1 (1.0 to 3.8)	2.2 (1.1 to 3.6)
**Weight gained by interpregnancy interval (%**, **99% CI)**				
0–11 months	65.3 (62.9 to 67.6)	61.7 (57.9 to 65.4)	62.4 (56.8 to 67.7)	61.0 (51.9 to 69.5)
12–23 months	60.3 (58.6 to 61.9)	60.3 (57.1 to 63.4)	62.8 (57.6 to 67.8)	63.1 (53.2 to 72.3)
24–35 months	66.2 (64.2 to 68.2)	64.5 (60.7 to 68.3)	60.8 (53.9 to 67.3)	73.7 (62.7 to 82.9)
36 months or more	74.0 (72.1 to 75.8)	72.2 (69.5 to 74.8)	72.0 (67.2 to 76.4)	79.0 (71.1 to 85.6)
**Change in maternal BMI category (%**, **99% CI)**				
No change in BMI category	71.6 (70.7 to 72.5)	71.2 (69.6 to 72.7)	69.6 (66.9 to 72.1)	69.4 (64.8 to 73.7)
Underweight (<18.5)	1.5 (1.3 to 1.8)	1.4 (1.0 to 1.8)	1.0 (0.5 to 1.7)	1.4 (0.5 to 2.9)
Normal weight (18.5 to 24.9)	45.1 (44.1 to 46.1)	39.1 (37.5 to 40.8)	34.0 (31.4 to 36.7)	30.4 (26.1 to 34.9)
Overweight (25.0 to 29.9)	13.6 (12.9 to 14.3)	15.2 (14.0 to 16.5)	15.7 (13.7 to 17.8)	15.7 (12.4 to 19.5)
Obese (≥30.0)	11.4 (10.9 to 11.9)	15.5 (14.3 to 16.7)	18.8 (16.7 to 21.1)	22.0 (18.2 to 26.1)
% decreased to normal weight	3.7 (3.3 to 4.1)	3.7 (3.1 to 4.4)	4.2 (3.2 to 5.5)	3.9 (2.3 to 6.2)
% decreased to overweight	1.5 (1.2 to 1.7)	2.5 (2.0 to 3.1)	2.2 (1.5 to 3.2)	2.6 (1.3 to 4.5)
% increased to overweight	11.7 (11.0 to 12.3)	11.5 (10.5 to 12.7)	11.2 (9.5 to 13.1)	12.5 (9.5 to 15.9)
% increased to obese	7.9 (7.4 to 8.5)	7.7 (6.8 to 8.6)	9.9 (8.3 to 11.6)	9.6 (7.0 to 12.8)

**Figure 2:**
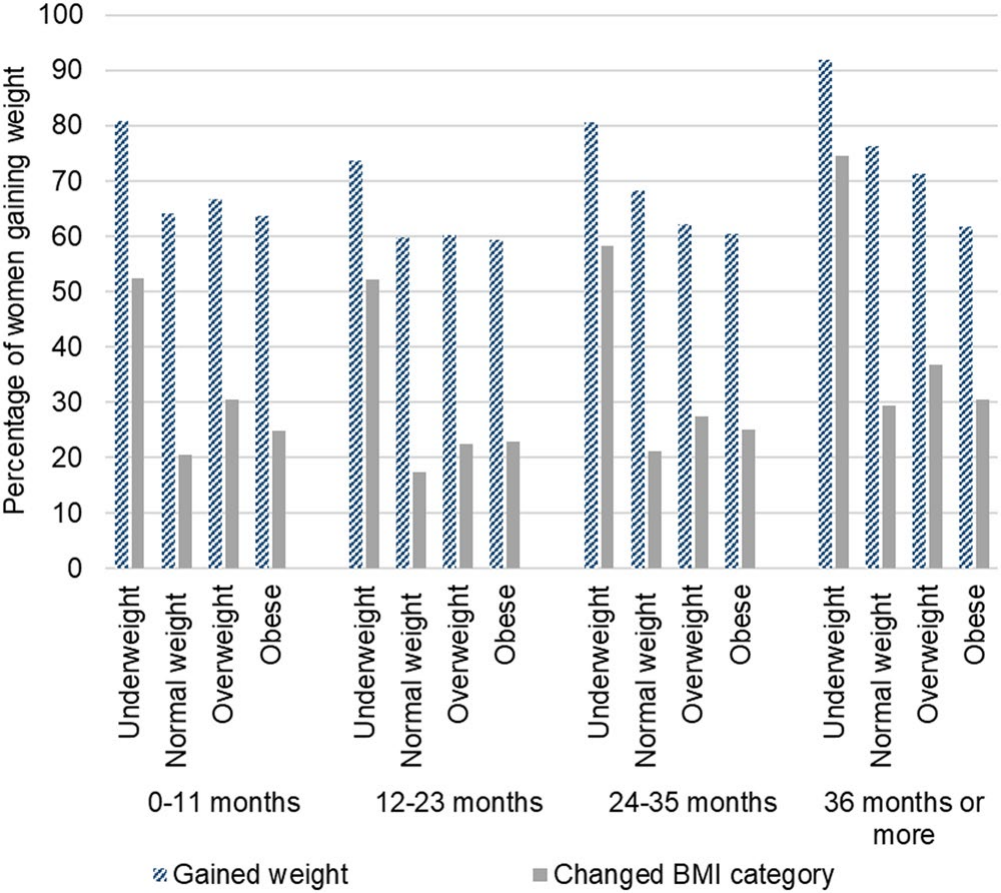
The percentage of weight gain by interpregnancy interval and maternal body mass index (BMI category) between first to second pregnancy.

Figure 2 shows the percentage of women gaining weight by BMI category and interpregnancy interval from first to second pregnancy. A substantial proportion of women within each BMI category gained weight across all intervals however, the lowest proportion of women gaining weight and changing BMI category across all BMI categories was in the 12–23 months interval. A similar pattern was observed across all pregnancies (data not presented).

**Figure 3:**
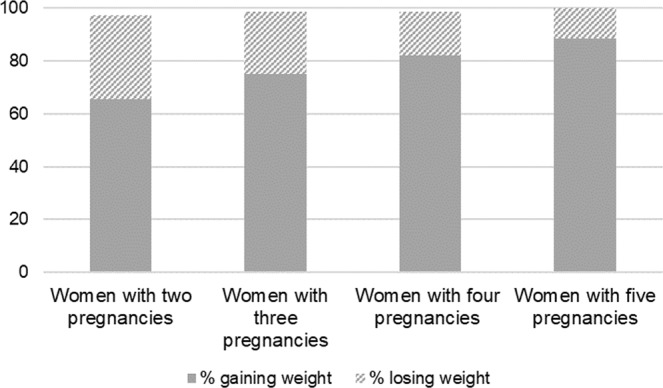
The percentage of weight gain and loss in women with two and more pregnancies across all their pregnancies.

Figure 3 summarizes the longer-term change in maternal BMI between pregnancies defined as the change in maternal BMI during the course of all her pregnancies in the dataset. The proportion of women who gained weight increased from 65.7% by second pregnancy in women who had their first two to 88.5% by fifth pregnancy in women who had their first five pregnancies.

In both unadjusted and adjusted linear regression analyses, there was a significant positive association between change in maternal BMI with each year of interpregnancy interval (adjusted increase in maternal BMI per year of interpregnancy interval 0.25 kg/m^2^, 99% CI 0.21 to 0.28) for first to second pregnancy. The coefficient remained similar across pregnancies and increased for the fourth to fifth pregnancy (adjusted increase in maternal BMI per year of interpregnancy interval 0.36 kg/m^2^, 99% CI 0.22 to 0.50) (Table 3).

**Table 3:** Linear regression estimates for association between change in maternal body mass index (BMI) measured at the first antenatal visit of each pregnancy and the length of the interpregnancy interval (in years).

	First to second pregnancy			Second to third pregnancy			Third to fourth pregnancy			Fourth to fifth pregnancy		
	n	Maternal BMI per year (99% CI)	p	n	Maternal BMI per year (99% CI)	p	n	Maternal BMI per year (99% CI)	p	n	Maternal BMI per year (99% CI)	p
Unadjusted	15940	0.27 0.23 to 0.30	<0.001	5738	0.22 0.17 to 0.27	<0.001	2165	0.24 0.16 to 0.32	<0.001	738	0.34 0.21 to 0.48	<0.001
Model 1	15940	0.27 0.24 to 0.31	<0.001	5738	0.22 0.18 to 0.27	<0.001	2165	0.25 0.17 to 0.33	<0.001	738	0.33 0.20 to 0.47	<0.001
Model 2	15259	0.25 0.21 to 0.28	<0.001	5498	0.24 0.19 to 0.29	<0.001	2081	0.25 0.16 to 0.33	<0.001	711	0.36 0.22 to 0.50	<0.001
Model 3	15259	0.25 0.21 to 0.28	<0.001	5498	0.24 0.19 to 0.29	<0.001	2081	0.25 0.16 to 0.33	<0.001	711	0.36 0.22 to 0.50	<0.001
Model 4	4667	0.17 0.07 to 0.26	<0.001	1608	0.19 0.04 to 0.33	0.001	617	0.07 −0.19 to 0.32	0.51	213	0.32 −0.06 to 0.71	0.03

Model 1 is adjusted for: timing of first (booking) antenatal appointments (as this is when maternal BMI is measured).

Model 2 is adjusted for: timing of first (booking) antenatal appointments, maternal age, ethnicity, highest educational qualification, whether undergone infertility treatment, smoking and employment status.

Model 3 is adjusted for: first (booking) antenatal appointment, maternal age, ethnicity, highest educational qualification, whether undergone infertility treatment, smoking, employment status and baseline maternal BMI (for the first pregnancy in the dataset).

Model 4 is adjusted for: first (booking) antenatal appointments, maternal age, ethnicity, highest educational qualification, whether undergone infertility treatment, smoking, employment status, baseline maternal BMI and breastfeeding or not at hospital discharge.

**Figure 4:**
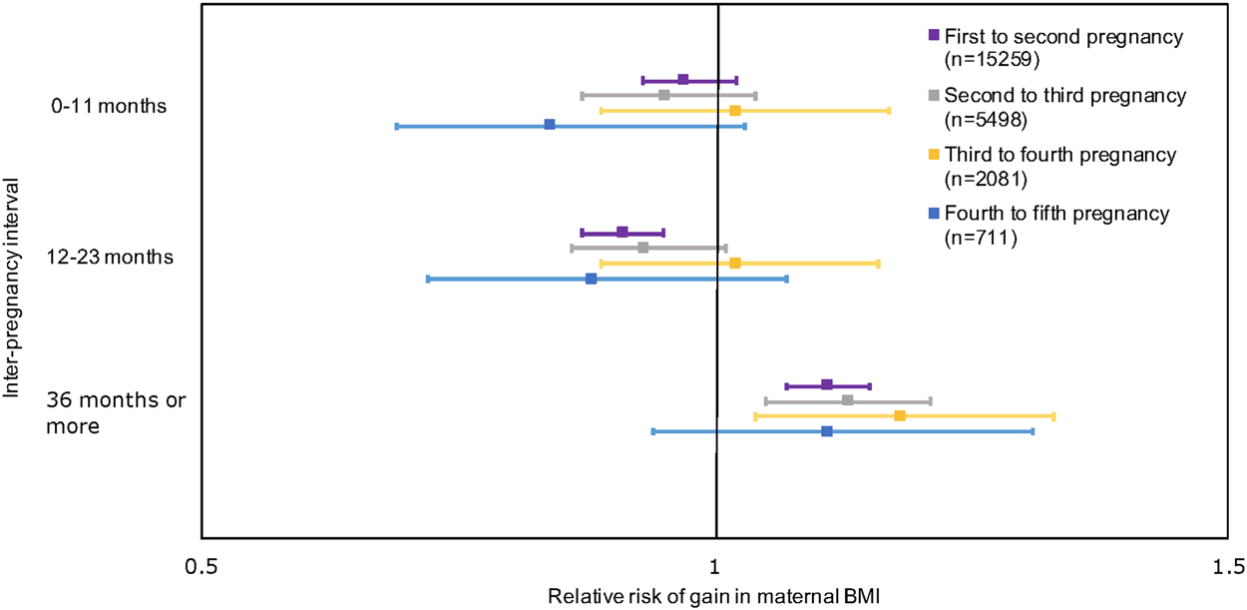
Adjusted association between interpregnancy gain in maternal body mass index (BMI) measured at the first antenatal visit of each pregnancy and the length of the interpregnancy interval (categorised).

The logistic regression models show that there is a significantly increased risk of starting the next pregnancy with a higher weight compared to the previous one with an interval of 36 months or more (adjusted RR 1.11, 99% CI 1.07 to 1.15 for first to second; adjusted RR 1.13, 99% CI 1.05 to 1.21 for second to third; adjusted RR 1.18, 99% CI 1.04 to 1.33 for third to fourth pregnancy) (Table 4, Fig. 4). In contrast, there was a significantly decreased risk of weight gain between pregnancies in those with an interval of 12 to 23 months (adjusted RR 0.91, 99% CI 0.87 to 0.95 for first to second; adjusted RR 0.93, 99% CI 0.86 to 1.01 for second to third; adjusted RR 1.02, 99% CI 0.89 to 1.16 for third to fourth pregnancy). The only exception was in women with five pregnancies where birth spacing was not significantly associated with interpregnancy weight gain in the period between their fourth and fifth pregnancies.

**Table 4:** Association between interpregnancy gain in maternal body mass index (BMI) measured at the first antenatal visit of each pregnancy and the length of the interpregnancy interval (categorised).

		Gain in maternal BMI: First to second pregnancy			Gain in maternal BMI: Second to third pregnancy			Gain in maternal BMI: Third to fourth pregnancy			Gain in maternal BMI: Fourth to fifth pregnancy		
		n	Relative risk (RR)* (99% CI)	p	n	RR (99% CI)	p	n	RR (99% CI)	p	n	RR (99% CI)	p
Unadjusted	0–11 m	15940	0.99 0.94 to 1.03	0.45	5738	0.96 0.88 to 1.04	0.16	2165	1.03 0.89 to 1.18	0.63	738	0.83 0.68 to 1.01	0.01
	12–23 m		0.91 0.87 to 0.95	<0.001		0.93 0.86 to 1.01	0.02		1.03 0.90 to 1.18	0.53		0.86 0.70 to 1.05	0.05
	24–35 m		(reference)			(reference)			(reference)			(reference)	
	>=36 m		1.12 1.07 to 1.16	<0.001		1.12 1.04 to 1.20	<0.001		1.18 1.04 to 1.34	<0.001		1.07 0.91 to 1.26	0.26
Model 1	0–11 m	15940	0.98 0.93 to 1.02	0.22	5738	0.95 0.87 to 1.03	0.13	2165	1.01 0.88 to 1.17	0.79	738	0.84 0.69 to 1.02	0.02
	12–23 m		0.91 0.87 to 0.95	<0.001		0.93 0.86 to 1.01	0.02		1.02 0.89 to 1.17	0.69		0.85 0.70 to 1.04	0.04
	24–35 m		(reference)			(reference)			(reference)			(reference)	
	>=36 m		1.12 1.08 to 1.16	<0.001		1.12 1.05 to 1.21	<0.001		1.19 1.05 to 1.34	<0.001		1.08 0.92 to 1.27	0.22
Model 2	0–11 m	15259	0.97 0.93 to 1.02	0.15	5498	0.95 0.87 to 1.04	0.13	2081	1.02 0.89 to 1.18	0.79	711	0.85 0.69 to 1.04	0.03
	12–23 m		0.91 0.88 to 0.95	<0.001		0.93 0.86 to 1.01	0.03		1.02 0.89 to 1.17	0.80		0.88 0.72 to 1.08	0.12
	24–35 m		(reference)			(reference)			(reference)			(reference)	
	>=36 m		1.11 1.06 to 1.15	<0.001		1.14 1.06 to 1.21	<0.001		1.18 1.04 to 1.34	0.001		1.11 0.94 to 1.31	0.11
Model 3	0–11 m	15259	0.97 0.93 to 1.02	0.14	5498	0.95 0.87 to 1.04	0.14	2081	1.02 0.89 to 1.17	0.70	711	0.84 0.69 to 1.03	0.03
	12–23 m		0.91 0.87 to 0.95	<0.001		0.93 0.86 to 1.01	0.02		1.02 0.89 to 1.16	0.76		0.88 0.72 to 1.07	0.09
	24–35 m		(reference)			(reference)			(reference)			(reference)	
	>=36 m		1.11 1.07 to 1.15	<0.001		1.13 1.05 to 1.21	<0.001		1.18 1.04 to 1.33	0.001		1.11 0.94 to 1.31	0.12
Model 4	0–11 m	4667	0.99 0.92 to 1.08	0.83	1608	0.93 0.81 to 1.07	0.17	617	0.96 0.76 to 1.22	0.68	213	0.78 0.60 to 1.03	0.02
	12–23 m		0.91 0.85 to 0.98	0.001		0.92 0.81 to 1.04	0.09		0.96 0.75 to 1.21	0.62		0.80 0.60 to 1.08	0.06
	24–35 m		(reference)			(reference)			(reference)			(reference)	
	>=36 m		1.12 1.03 to 1.21	0.001		1.08 0.95 to 1.24	0.13		0.99 0.76 to 1.31	0.96		1.00 0.77 to 1.31	0.97

^*^Generalised linear model with log link and robust variance estimator used to derive RR.

Model 1 is adjusted for: timing of first (booking) antenatal appointments (as this is when maternal BMI is measured).

Model 2 is adjusted for: timing of first (booking) antenatal appointments, maternal age, ethnicity, highest educational qualification, whether undergone infertility treatment, smoking and employment status.

Model 3 is adjusted for: first (booking) antenatal appointment, maternal age, ethnicity, highest educational qualification, whether undergone infertility treatment, smoking, employment status and baseline maternal BMI (for the first pregnancy in the dataset).

Model 4 is adjusted for: first (booking) antenatal appointments, maternal age, ethnicity, highest educational qualification, whether undergone infertility treatment, smoking, employment status, baseline maternal BMI and breastfeeding or not at hospital discharge.

## Discussion

This study examined the association of change in maternal BMI between pregnancies with interpregnancy interval in 19362 women in Hampshire, England. The rate of obesity increased from 13.0% at first pregnancy to 31.6% at fifth pregnancy, with approximately two thirds of the study sample gaining weight by the start of their subsequent pregnancy compared to the start of their previous one. An interval of 12 to 23 months between the first and second pregnancy was found to confer the lowest risk of weight gain, and hence of starting the next pregnancy with a higher weight. This association remained statistically significant after adjusting for maternal age and starting maternal BMI.

About 22% of women presented to antenatal care for their subsequent pregnancy in a higher BMI category, compared to 4–6% in a lower BMI category than the previous pregnancy. These findings are comparable to those from a previous study of a longitudinal cohort in Dublin^13^. Only two percent of women in a higher BMI category at the start of a subsequent pregnancy were underweight at the previous pregnancy and so had moved up into the healthier category of normal weight. An additional eight percent of women were obese at the start of a subsequent pregnancy with this rising to 10% in higher order (fourth and fifth) pregnancies. This pattern of weight gain was seen across pregnancies and thus we additionally show that this persists through subsequent pregnancies and not just from the first to second.

Relatively small BMI gains (1–2 units) increases the risk of perinatal complications in the subsequent pregnancy even if the woman remains normal weight^23^. In this sample, women changed one BMI unit between pregnancies on average whereas in the two-thirds that gained weight the average gain was two BMI units with some women gaining substantially more. The proportions of overweight and obesity in this sample were higher in subsequent pregnancies compared to the first. It is not possible to attribute weight change between pregnancies purely to pregnancy related factors but with two-thirds of the women in this cohort gaining weight and under a third losing weight, the likelihood is that pregnancy plays an influential role in this weight change, particularly given the small percentage (2.5%) whose weight did not change.

To our knowledge, this is the first cohort study investigating the association between birth spacing and maternal weight change between pregnancies. The study sample is based on a relatively large population-based cohort including women from all socioeconomic backgrounds, thus representative of the regional population. One city may not be representative of the general population of the country and according to the UK Department of Communities and Local Government English indices of deprivation report, Southampton is more deprived than average with the situation having worsened between 2010 and 2015^24^. However, about half of the women included in this analysis reside in surrounding areas to Southampton in Hampshire, many of which are much less deprived. The sample was 87% White comparable to the 2011 England and Wales population census of 86% White^25^. The analysis was adjusted for several key confounders that were reasonably complete (96% complete for ethnicity and employment status).

An important limitation was the lack of information on weight gain during pregnancy, which is a key factor influencing post-partum weight. Women who had their first booking appointment later into the pregnancy (more than 24 weeks) were excluded from the analysis in order to ensure comparability of weight measurements between pregnancies. BMI was measured in early pregnancy at the booking appointment at a median of 11 weeks, however 13–21% of women across the pregnancies were measured between 14 to 24 weeks of pregnancy and thus weight could be slightly overestimated which is why timing of booking appointment was adjusted for in all analyses. Breastfeeding initiation and duration can also influence post-partum weight. No information was available on breastfeeding duration and although breastfeeding initiation (at discharge) was available, this was only recorded in a little over a third of the pregnancies included. Another limitation is that these findings are based on observational data so inferences about causation cannot be drawn and the risk of residual confounding influencing the results needs to be considered. However, it is not feasible or ethical to conduct a randomised trial to address the aim of this study.

To our knowledge, the only international guideline on birth spacing is the 2005 WHO technical consultation published in 2007 which recommends waiting at least 24 months after a previous live birth^14^. This was based on evidence on maternal, perinatal, infant and child health outcomes from a wide range of countries. However, in light of the rising rates of maternal obesity and its consequences on pregnancy outcomes and maternal and offspring health, updated recommendations on the optimal interpregnancy interval would benefit from incorporating evidence around this such as that generated by this study. A shorter optimal interval is further supported by the findings of a meta-analysis of 62 studies that an interpregnancy interval of 18 to 23 months was associated with the lowest risk of adverse perinatal outcomes in the offspring with both shorter (<18 months) and longer (>59 months) intervals being associated with increased risk^20^.

A qualitative study in Sweden in women who had retained ≥10 kg postpartum found that the first year postpartum is a neglected year in women with the focus of care being on the baby with little or no weight loss support. The main areas identified related to weight retention were a lack of knowledge, misconceptions, eating for relief, lack of support and barriers to physical activity including tiredness and competing responsibilities^26^. Another study reported that women considered their personal health was not top priority during the early postpartum period and identified childcare, time management and lack of support as barriers to adopting healthier lifestyles^27^. Lifestyle changes were motivated by child’s health in women diagnosed with gestational diabetes during pregnancy with vague understanding and low levels of concern of increased future risk of Type 2 diabetes^28^. Another study in Sweden also found that a healthier lifestyle adopted during pregnancy and in early parenthood was motivated by supporting a health-promoting environment for the child^29^ and thus weight retention in the context of the health of future children could be a motivator to promoting weight loss.

Stabilizing interpregnancy weight and promoting weight loss in overweight and obese women before the next pregnancy could be important steps in reducing adverse outcomes in subsequent pregnancies. The use of the six to eight week postnatal check to discuss women’s weight is part of the National Institute for Health and Care Excellence guidelines^30^. However, only women with a pre-pregnancy BMI of 30 kg/m^2^ or more are recommended to have a discussion with their health professional about the increased risk of being obese and encouraged to lose weight, particularly that gained during pregnancy. Additionally, the interpregnancy interval is not discussed as there are no UK guidelines on interval. The health and wellbeing of the mother needs to be considered with an equal focus as to the health of the baby for any preventive measures during the period between pregnancies. More research is needed, considering other short and long-term maternal and offspring outcomes, to investigate the optimal interpregnancy interval in high-income countries.

In conclusion, most women do not maintain their weight across pregnancies, with substantially more gaining than losing weight. An interpregnancy interval of 12–23 months was associated with the lowest risk of starting the second pregnancy with a higher body weight compared to the start of the previous pregnancy. Preventing weight gain and continuing to support weight loss in overweight and obese women between pregnancies are important preventive measures of subsequent adverse maternal and offspring health outcomes. Further research investigating optimal birth spacing in relation to important public health risk factors such as maternal and childhood obesity is needed.

## Methods

This is a population-based cohort of prospectively collected routine healthcare data for antenatal care between January 2003 and September 2017 at University Hospital Southampton, Hampshire, UK. This included all women delivering at this hospital, which is a regional centre for maternity care in and around Southampton. Records of women with two or more consecutive singleton live birth pregnancies were included. Analysis was carried out by pregnancy order by using information on parity to categorise the pregnancies as first to second, second to third, third to fourth and fourth to fifth, even if the previous births were not recorded in the analysed dataset (e.g. if the woman had received antenatal care elsewhere). Women with more than five previous births (due to small numbers) and records with unfeasible weight, height and gestational age values were excluded. Only singleton pregnancies were included.

### Exposure assessment

The difference in days between two consecutive live births was calculated and gestational age of the latter birth subtracted from this to derive the interpregnancy interval. For multiparous women, no information was available on the interval from a previous pregnancy if delivery was before the start of the study period (2003) or at another hospital. Only women whose pregnancies resulted in live births were included as other pregnancy outcomes (stillbirth, miscarriage) could affect the interpregnancy interval^31^. A categorical variable with categories of 0–11, 12–23, 24–35 and 36 months or more was created. The 24–35 month category was used as the reference category as this was in line with the World Health Organization guideline of at least 2 years^14^.

### Outcome assessment

Maternal weight in kilograms was measured at the first antenatal (booking) appointment of each pregnancy, which is recommended ideally by 10 weeks gestation in the UK^32^. The booking appointment is booked by midwives once pregnancy is confirmed by general practice. Women are prioritised by gestational age with the aim of booking the appointment during the recommended period. Any woman who had a booking appointment at or after 24 weeks of pregnancy was excluded. BMI was calculated as weight (in kg) divided by height (in metres) squared. BMI was analysed as both a continuous (kg/m^2^) and categorical variable. The categorical variable was defined as underweight (BMI < 18.5 kg/m^2^), normal weight (18.5 to 24.9 kg/m^2^), overweight (25.0 to 29.9 kg/m^2^) and obese (≥30 kg/m^2^). Change in BMI was calculated as the difference in BMI measured at booking appointment between two consecutive live birth pregnancies. Weight gain was calculated as any gain in weight that led to a change in BMI between the two measurement points. Baseline BMI was defined as the BMI at the first pregnancy that information was available for.

Gestational age (date of last menstrual period) is discussed and recorded at the booking appointment. Gestational age at birth is determined based on an ultrasound-dating scan which usually takes place after the booking appointment.

### Covariates

Maternal date of birth is recorded at the booking appointment and converted to age on extraction of the dataset to maintain anonymity. Highest maternal educational qualification was self-reported and categorised as primary, secondary, college, undergraduate, postgraduate, graduate and none. For the purposes of this analysis, this was condensed to three categories - secondary (GCSE) and under, college (A levels) and university degree or above. Self-reported ethnicity was recorded under 16 categories and condensed to White, Mixed, Asian, Black/African/Caribbean, Chinese and Other. Categories of not asked and not stated were coded as missing. Smoking was self-reported as current smoking or non-smoking. Non-smokers were further asked if they had ever smoked or had previously smoked and quit. This was categorised as stopped more than 12 months before conception, stopped less than 12 months before conception or stopped when pregnancy confirmed. Employment was self-reported at booking appointment and categorised as employed, unemployed, in education, and not specified. Infertility treatment was categorised as no/investigations only and yes (hormonal only, *in-vitro* fertilisation, gamete intrafallopian transfer and other surgical) in either one or both pregnancies. Breastfeeding was recorded at discharge from the hospital as exclusive, partial or no breastfeeding.

### Ethical approval

All data were anonymised to the research team. Ethics approval was granted by the University of Southampton Faculty of Medicine ethics committee: study id 25508 on 14/06/2017. All research was performed in accordance with relevant guidelines and regulations.

### Statistical analysis

All analysis was performed using Stata 15^33^. Linear regression was used to examine the association of maternal change in BMI between pregnancies (assessed as a continuous variable in kg/m^2^) with interpregnancy interval (assessed as a continuous variable in years). Generalised linear regression with log link and robust variance estimator^34^ was then used to examine the same association (maternal change in BMI with interpregnancy interval) but by categorising maternal change in BMI into gained weight compared with no change or lost weight using the detailed categorisation of interpregnancy interval described above.

Initial univariable analysis was followed by multivariable models adjusting for potential confounding factors – timing of booking appointment (as this is when BMI is measured), maternal age, ethnicity, highest educational qualification, whether or not undergone infertility treatment, employment status, smoking behaviour and baseline maternal BMI. Finally, the role of a potential mediating factor (breastfeeding behaviour at hospital discharge) was examined in the subgroup in which this data was available.

A statistical significance level of 0.01 with 99% confidence intervals was used in the regression models to reduce the risk of Type I error due to multiple testing.

## Data Availability

The authors’ ethical approval from the Faculty of Medicine Ethics Committee, University of Southampton (Reference number 25508) restricts public sharing of the data used in this study. Please contact the authors to request data access beyond that included in the manuscript. Further ethical and research governance approval may be required.
